# Binding and neutralizing anti-AAV antibodies: Detection and implications for rAAV-mediated gene therapy

**DOI:** 10.1016/j.ymthe.2023.01.010

**Published:** 2023-01-11

**Authors:** Martin Schulz, Daniel I. Levy, Christos J. Petropoulos, George Bashirians, Ian Winburn, Matthias Mahn, Suryanarayan Somanathan, Seng H. Cheng, Barry J. Byrne

**Affiliations:** 1 Pfizer, 235 East 42nd Street, New York, NY 10017, USA; 2 Labcorp-Monogram Biosciences, South San Francisco, CA 94080, USA; 3 University of Florida, 1600 SW Archer Road, Gainesville, FL 32610, USA

**Keywords:** recombinant adeno-associated virus, gene therapy, neutralizing antibodies, seroprevalence, efficacy, safety, transduction inhibition assay, total antibody assay, humoral immunity

## Abstract

Assessment of anti-adeno-associated virus (AAV) antibodies in patients prior to systemic gene therapy administration is an important consideration regarding efficacy and safety of the therapy. Approximately 30%–60% of individuals have pre-existing anti-AAV antibodies. Seroprevalence is impacted by multiple factors, including geography, age, capsid serotype, and assay type. Anti-AAV antibody assays typically measure (1) transduction inhibition by detecting the neutralizing capacity of antibodies and non-antibody neutralizing factors, or (2) total anti-capsid binding antibodies, regardless of neutralizing activity. Presently, there is a paucity of head-to-head data and standardized approaches associating assay results with clinical outcomes. In addition, establishing clinically relevant screening titer cutoffs is complex. Thus, meaningful comparisons across assays are nearly impossible. Although complex, establishing screening assays in routine clinical practice to identify patients with antibody levels that may impact favorable treatment outcomes is achievable for both transduction inhibition and total antibody assays. Formal regulatory approval of such assays as companion diagnostic tests will confirm their suitability for specific recombinant AAV gene therapies. This review covers current approaches to measure anti-AAV antibodies in patient plasma or serum, their potential impact on therapeutic safety and efficacy, and investigative strategies to mitigate the effects of pre-existing anti-AAV antibodies in patients.

## Graphical abstract

**Figure:**
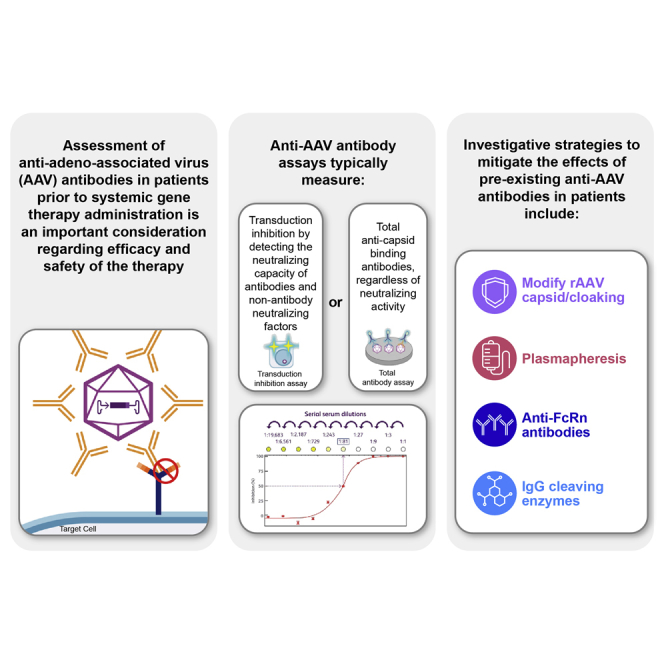

## Teaser

Assessment of anti-adeno-associated virus (AAV) antibodies in patients prior to systemic gene therapy administration is an important consideration regarding efficacy and safety of the therapy. This report reviews the approaches to measure anti-AAV antibodies, their potential impact on therapeutic outcomes, and investigative strategies to mitigate their effects in patients.

## Introduction

Current *in vivo* gene therapy (GTx) approaches primarily focus on rare monogenic disorders caused by loss-of-function or pathogenic gain-of-toxic-function mutations, and typically involve recombinant viral vector delivery of a therapeutic gene.^1^^,^^2^ The most commonly used viral vectors for GTx are based on adeno-associated virus (AAV), a non-enveloped single-stranded DNA virus.^3^ AAV is a member of the *Dependoparvovirus* genus of the Parvoviridae family.^4^^,^^5^ Wild-type AAV is considered non-pathogenic, clinically silent,^6^^,^^7^ and requires co-infection with a helper virus (adenovirus or herpesvirus) to facilitate replication.^6^^,^^7^ The capsid of AAV comprises an assembly of three structural proteins (VP1–VP3) in the ratio of 1:1:10 (VP1:VP2:VP3).^8^^,^^9^ The packaged recombinant AAV (rAAV) vector genome lacks all viral genes, but instead includes the transgene of interest,^4^ plus regulatory elements that promote efficient and targeted transgene expression.^8^ AAV serotypes differ in their tissue tropism^10^^,^^11^; therefore, the selection of an appropriate AAV serotype further enables targeted tissue expression.^4^^,^^10^^,^^12^ It is estimated that about 30%–60% of the population have measurable antibodies to different AAV serotypes from a wild-type infection.^13^ These antibodies can potentially inhibit the transduction of target cells by rAAV vectors, thus impeding successful gene transfer, and may have potential safety consequences.^13^ Several studies have demonstrated that anti-AAV antibody seroprevalence in humans varies geographically^14^^,^^15^ and with age.^15^^,^^16^^,^^17^^,^^18^ Furthermore, owing to the high degree of conservation in the capsid amino acid sequence, substantial cross-reactivity exists among AAV serotypes.^13^^,^^14^^,^^15^^,^^19^ Separate from wild-type exposure, there is the theoretical risk that shedding of rAAV vectors in bodily fluids by patients who received rAAV GTx might lead to seroconversion of household members and other close contacts.^20^ Also, with ongoing clinical research for rAAV-based vaccines, there is concern that recipients of these vaccines might develop antibodies against AAV, which could render them ineligible for future rAAV GTx.^21^^,^^22^ Both of these potential risks require additional investigation.

Evaluating anti-AAV antibodies is considered essential before the administration of systemic rAAV GTx, and pre-existing anti-AAV antibodies above a predetermined threshold are currently an accepted exclusion criterion in many rAAV GTx clinical trials.^23^^,^^24^^,^^25^ This review discusses current approaches to measuring anti-AAV antibodies in patient plasma or serum, the potential safety and efficacy consequences associated with the presence of these antibodies, and investigative strategies to mitigate the effects of pre-existing anti-AAV antibodies in patients. This review focuses on the challenges associated with pre-existing antibodies, as opposed to antibodies developed after systemic rAAV GTx administration.^26^ The time course and the magnitude of antibody formation after GTx involves several considerations that are not part of this review.

### Overview of anti-AAV antibodies

**Figure 1:**
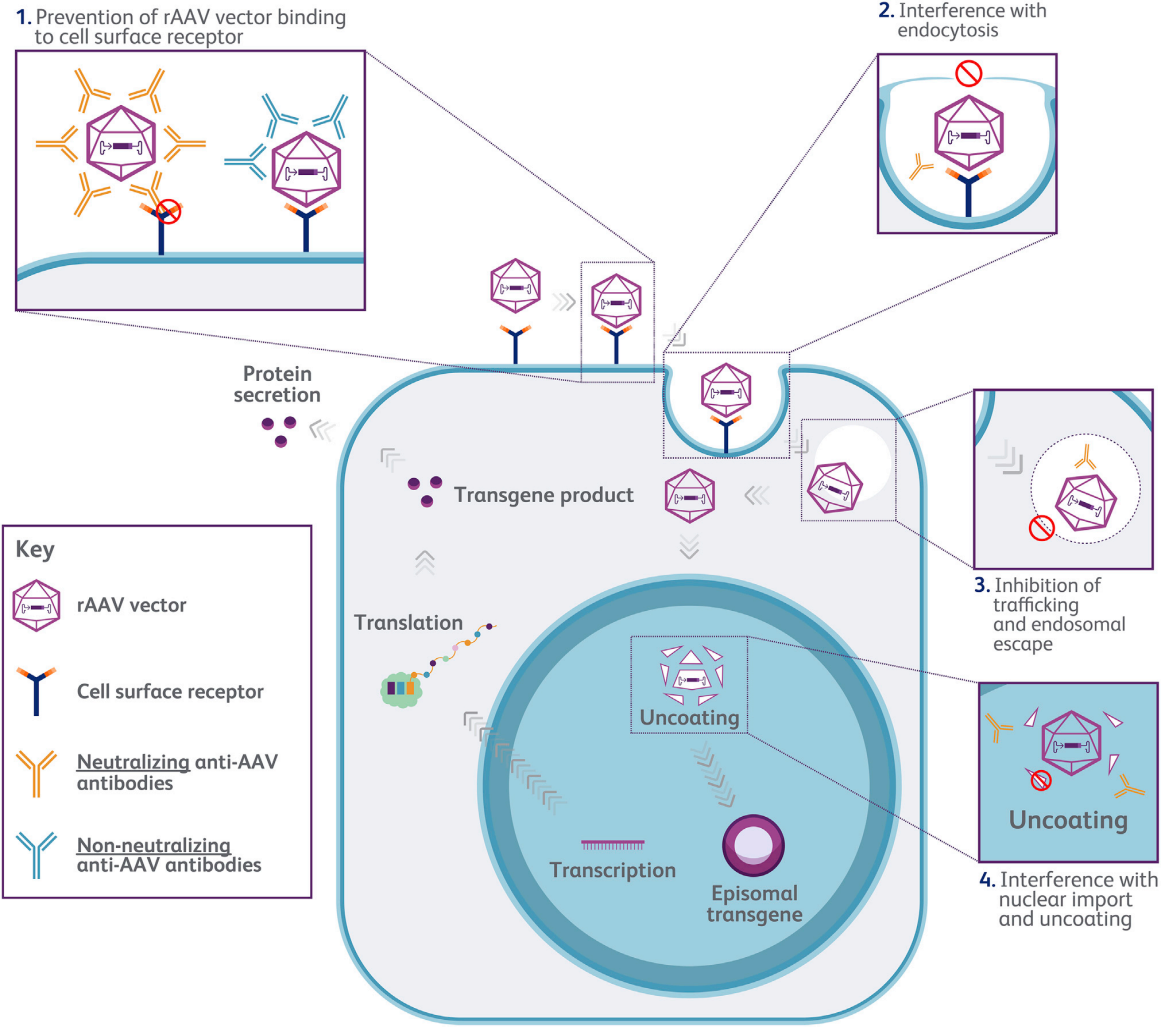
Mechanisms for the inhibition of vector transduction and transgene expression by neutralizing anti-AAV antibodies rAAV vector transduction and key steps in which transduction may be inhibited by neutralizing anti-AAV antibodies. Non-neutralizing anti-AAV antibodies bind to the rAAV capsid but do not prevent vector binding to cell-surface receptors or inhibit transduction. Of note, non-antibody neutralizing factors (not depicted) might also impact transduction. AAV, adeno-associated virus; rAAV, recombinant AAV.

Anti-AAV antibodies can be neutralizing or non-neutralizing; their potential impact on vector transduction is illustrated in Figure 1. Neutralizing antibodies (NAbs) generally bind to the rAAV capsid and can inhibit vector transduction, while non-neutralizing antibodies (non-NAbs) bind to the AAV capsid but do not impede vector transduction (in some cases, they may even enhance AAV transduction).^27^ Both NAbs and non-NAbs can potentially impact biodistribution of the vector away from target cells by retargeting it to secondary lymphoid organs.^27^ Non-systemic, direct administration of rAAV vectors into immune-privileged sites, including the eye or the central nervous system, may be less affected by anti-AAV antibodies.^28^^,^^29^^,^^30^

Anti-AAV antibody seroprevalence is higher for a few months after birth due to active maternal transfer of antibodies, known as passive immunity.^31^^,^^32^ In late infancy, seroprevalence remains low until around 3 years of age, after which it progressively increases throughout childhood and adulthood as a result of wild-type AAV exposure.^17^^,^^18^ During an individual’s life, anti-AAV antibody levels may remain stable or fluctuate over time. A study investigating NAb levels in chimpanzees found that subsets of animals were seronegative, seropositive, or seroconverted with fluctuating titers over the 10-year observational period.^14^ Seroconversion of anti-AAV antibody-negative patients in the period between the determination of treatment eligibility and GTx administration is a concern, although the extent to which this might actually happen is currently unknown. A longitudinal study of healthy donors (N = 30) over 3 years found that AAV8 NAb titers were stable and seroconversion was infrequent and limited to donors with borderline positive-negative titers.^19^ It is anticipated that GTx clinical trials involving run-in periods, in which patients are tested for antibodies at regular periods, will provide additional data on this issue. A randomized phase 3 Duchenne muscular dystrophy (DMD) GTx trial, for example, includes a cohort of patients who will be dosed 1 year after enrollment, generating longitudinal seroprevalence data.^33^

**Figure 2:**
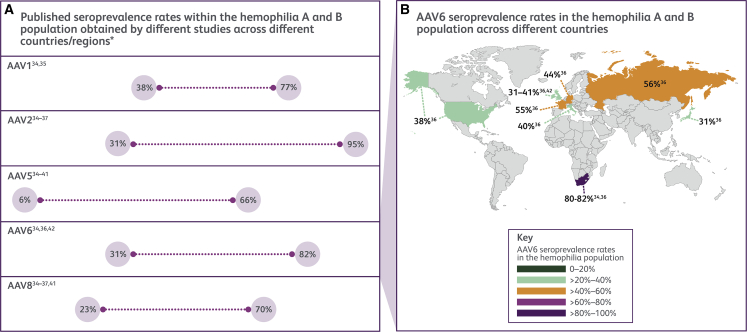
Anti-AAV antibody seroprevalence ranges for different AAV serotypes and across geographical regions using the hemophilia A and B population as an example The published anti-AAV antibody seroprevalence rates vary widely according to a variety of factors, including (A) different AAV serotypes,^18^^,^^34^^,^^35^^,^^36^^,^^37^^,^^38^^,^^39^^,^^40^^,^^41^^,^^42^ and (B) across geographical regions (using AAV6 data from three separate reports as an example).^34^^,^^36^^,^^42^ Other factors that may influence seroprevalence rates include assay type, donor health and age, and the use of some medications, such as immunosuppressants. ∗As these studies were performed in different regions/countries using different assay types, the seroprevalence rates cannot be directly compared. AAV, adeno-associated virus; TAb, total antibody; TI, transduction inhibition.

Anti-AAV antibody seroprevalence depends on numerous factors, such as AAV capsid serotype, age, assay type (Figure 2A),^18^^,^^34^^,^^35^^,^^36^^,^^37^^,^^38^^,^^39^^,^^40^^,^^41^^,^^42^ and geographical location (Figure 2B).^34^^,^^36^^,^^42^ Other factors that may affect seroprevalence include donor health (healthy or diseased)^19^^,^^37^^,^^40^^,^^41^^,^^43^^,^^44^ and the use of immunosuppressants, such as rituximab and cyclosporine.^13^^,^^45^ Substantial cross-reactivity of anti-AAV antibodies between the serotypes is evident,^46^ which is most likely explained by the high degree of AAV capsid sequence homology.^13^^,^^19^^,^^47^ Kruzik et al. (2019) found that 87% of individuals with NAbs directed against multiple AAV serotypes exhibited higher titers against AAV2, suggesting that the lower titers against AAV5 and AAV8 in the same sample may be due to cross-reactivity of anti-AAV2 NAbs.^19^ Future studies interrogating the amino acid or conformational motifs driving cross-reactive anti-AAV antibody responses are needed to improve our understanding of the variations in immunogenicity across serotypes.

### Assays to screen for anti-AAV antibodies

Several methods have been developed to detect anti-AAV antibodies prior to GTx administration. To date, transduction inhibition (TI) assays and total antibody (TAb) assays have been routinely used in clinical trials.^48^^,^^49^ These assays typically use patient plasma or serum samples, but for the remainder of this text such samples will be referred to as serum samples for simplicity reasons.^48^^,^^49^ Clinical trial registration documentation and prescribing information for rAAV GTx may include the assay design and the threshold of the anti-AAV antibody titer to determine patient eligibility (screening titer cutoff), which varies from study to study.^50^^,^^51^^,^^52^^,^^53^^,^^54^^,^^55^

### TI assays

**Figure 3:**
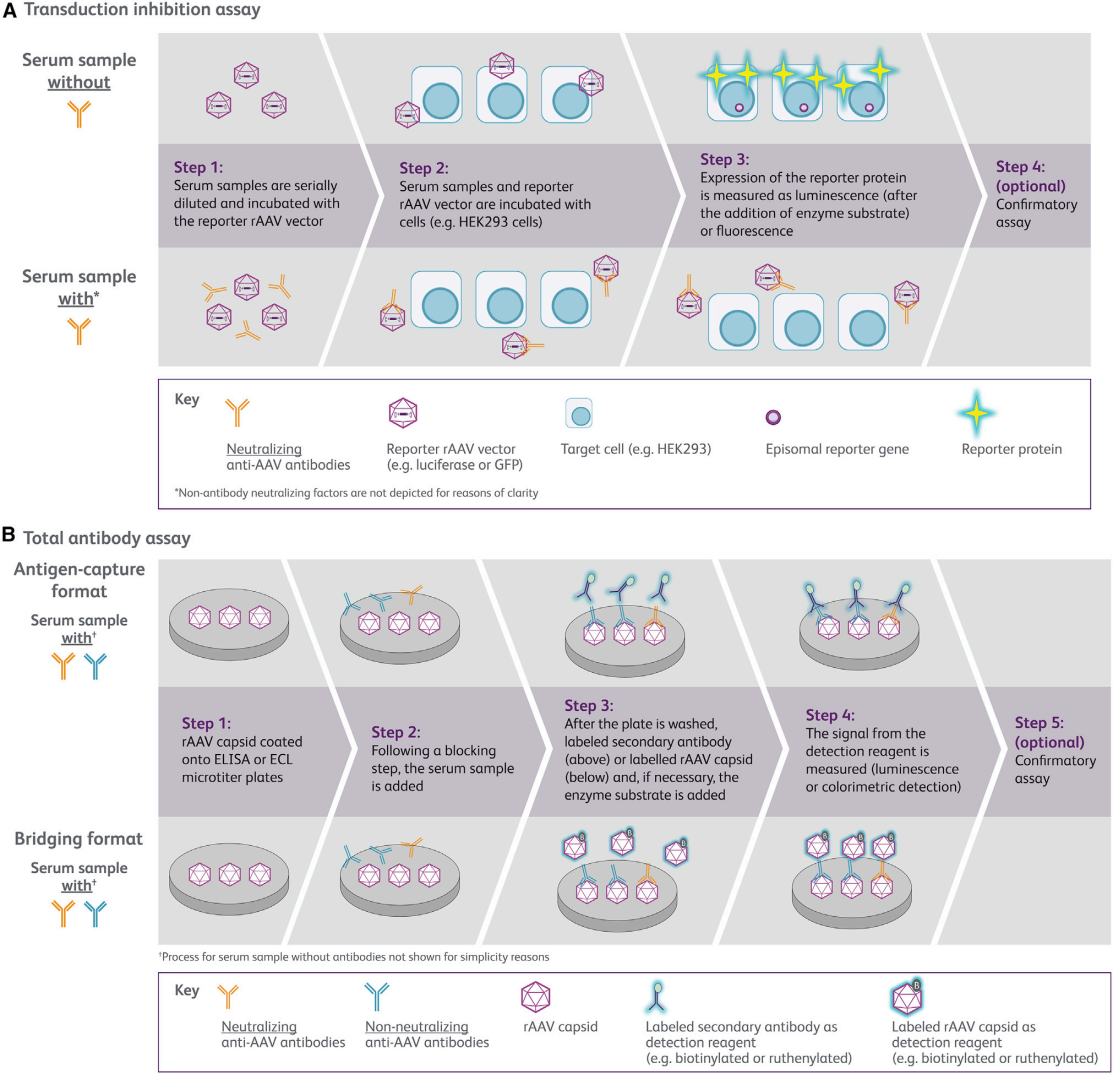
Principles of transduction inhibition and total antibody assays (A) (1) Serum samples are heat-inactivated and any potential precipitates removed by centrifugation. Patient and control serum dilutions are prepared. The TI assay is usually carried out in a 48- or 96-well plate format, permitting a high-throughput sample analysis. Typically, a reporter rAAV vector is combined with the serially diluted test sample before (2) being incubated with the cell line (in some cases, target cells are pre-infected with wild-type adenovirus to increase rAAV transduction).^14^ (3) The target cells are lysed, and reporter gene expression (luciferase or GFP activity) is measured as luminescence after the addition of the enzyme substrate (in the case of luciferase) or fluorescence (in the case of GFP). The presence of AAV NAbs and non-antibody neutralizing factors (not depicted in the figure for reasons of clarity) interferes with the transduction process and decreases the reporter gene expression when compared with the negative control. (4) Confirmatory steps to determine neutralization due to NAbs can be performed, although they are not always essential. This step may involve use of an irrelevant monoclonal non-AAV antibody to determine specificity, Ig fraction depletion, or competitive inhibition with empty vectors (or irrelevant transgenes).^49^ (B) TAb assays can be divided into antigen-capture and bridging formats, depending on the secondary reagents used. TAb assays can be developed using either co-incubation (homogeneous) or sequential incubation (heterogeneous) protocols based on the desired attributes, such as improved assay selectivity, antigen tolerance, or specificity (reviewed in Gorovits et al.^48^). (1) rAAV capsids are immobilized on an ELISA or electrochemiluminescence (ECL) microtiter plate; (2) serum samples are added to allow binding of antibodies to the rAAV capsid; (3) after washing to remove unbound material, in the antigen-capture format, a secondary detection reagent such as a horseradish peroxidase-conjugated or ruthenylated anti-species antibody is added; in the bridging format, a labeled (e.g., biotinylated or ruthenylated) rAAV capsid is added^48^). (4) In the case of a ruthenylated detection system, the assay signal is measured in luminescence units after the addition of the read buffer. For enzyme-conjugated detection systems, the enzyme substrate is added for colorimetric detection. Commercially available serotype-specific anti-AAV capsid monoclonal/polyclonal antibodies or proprietary antibodies may be used as a positive control, while pooled samples from TAb-negative donors can be used as a negative control.^48^ (5) Anti-AAV TAb screening assays typically involve an additional confirmatory assay to ensure the specificity of the signal obtained. AAV, adeno-associated virus; ECLA, electrochemiluminescence assay; ELISA, enzyme-linked immunosorbent assay; GFP, green fluorescent protein; Ig, immunoglobulin; NAb, neutralizing antibody; rAAV, recombinant AAV; TAb, total antibody; TI, transduction inhibition.

TI assays, generally referred to and understood as NAb assays, measure the extent to which NAbs and non-antibody neutralizing factors inhibit the rAAV-mediated expression of a reporter gene.^48^ Using a cell-based approach, the TI assay uses the same capsid employed in the particular GTx, but contains a “reporter” gene for ease of detection (Figure 3A).^23^^,^^48^ The reporter vector construct needs to be produced and purified in a similar way as the corresponding GTx vector to ensure both have comparable analytics (empty:full capsid ratio, percentage of intact genomes, impurities) that may otherwise affect assay results. Patient serum is serially diluted and pre-incubated with the rAAV reporter vector before inoculating target cells.^49^ Following an incubation period to allow for target cell transduction and expression of the reporter gene, the rAAV neutralizing titer is assessed by measuring the reduction in expression of the reporter gene.^49^ The neutralizing titer is usually defined as the highest serum dilution that reduces rAAV transgene expression by a specified amount (e.g., ≥50%) or the extrapolated dilution derived from an inhibition curve (ID_50_).^49^^,^^56^ TI assays measure the presence of NAbs as well as non-antibody neutralizing factors that inhibit transduction. Non-antibody neutralizing factors may include small molecules, innate immune activators, and shed AAV receptors.^49^^,^^57^^,^^58^^,^^59^^,^^60^ Although not yet fully understood, NAbs and non-antibody neutralizing factors are thought to inhibit transduction and transgene expression via one or more mechanisms that include blocking vector uptake into target cells, preventing endosomal escape, obstructing capsid uncoating, and impeding nuclear entry (Figure 1).^49^^,^^61^

rAAV reporter vectors typically contain genes that provide convenient and sensitive detection of transduction, such as green fluorescent protein (GFP), β-galactosidase, or luciferase, instead of the therapeutic transgene.^23^^,^^49^ To assess neutralizing activity directed against the rAAV, the expression of the reporter gene is measured in the presence and absence of the test sample along with positive-control (PC) and negative-control (NC) samples. The ability of the assay to detect low-titer NAbs may depend on the specific characteristics of the reporter gene, the AAV capsid, and the assay design. Multiple studies have reported more sensitive NAb detection using a TI assay with a luciferase reporter system versus a GFP reporter.^40^^,^^62^^,^^63^ As an alternative to using a reporter gene, expression levels of the messenger RNA (mRNA) encoded by the therapeutic transgene may be used as an endpoint in a similar manner to assays used for potency assessment in rAAV GTx batch-release testing.^49^

Various cell types have been used for TI assays, including HEK293, HeLa, and Huh7 cell lines,^14^^,^^49^^,^^64^ with HEK293 being the most widespread.^49^ Different rAAV capsid serotypes vary in their transduction efficiencies for these cell lines. Poorly transducing capsids resulting in lower transgene expression require higher MOIs (multiplicity of infection, i.e., the ratio of vector particles to a target cells) or transduction enhancing reagents (wild-type adenovirus,^65^ ecdysone induced adenovirus proteins,^56^ compound C^66^) to achieve measurable transgene expression. However, the use of higher MOIs modifies the stoichiometry of antibodies to capsids that can impact assay results.^67^ Other factors that may influence the assay results include the sample matrix, sample starting dilution, target cell number, assay incubation time and temperature, the volume of serum used, and heat inactivation of complement proteins.^63^ The use of reagents such as heparin, an anti-coagulant commonly used for blood collection, may also influence the transduction of HeLa or HEK293 cells through dose-dependent, competitive binding to proteoglycans.^49^^,^^68^ Factors related to sample collection and processing may also impact results, including sample-collection devices/tubes, sample integrity (hemolysis, lipemia), sample handling, and storage conditions.^62^

### TAb assays

TAb assays, also known as binding antibody assays, measure all capsid-bound antibodies whether or not they have neutralizing (inhibitory) activity (Figure 1).^23^^,^^24^ As TAb assays only detect antibodies,^48^ they do not measure non-antibody neutralizing factors.

TAb assays typically involve coating rAAV vectors (full or empty) or AAV peptides onto a plate (Figure 3B). They can detect all anti-AAV antibody isotypes,^48^ including those of low avidity that may not have clinical relevance.^69^ These assays can also detect the predominance of anti-AAV immunoglobulin (Ig) using class-specific secondary antibodies.^48^ There is substantial variation among conventional TAb assay platforms. An antigen-capture assay evaluates anti-AAV antibodies that directly bind to the capsid using an enzyme-linked immunosorbent assay (ELISA) or electrochemiluminescence assay (ECLA). Isotype detection for antigen-capture assays relies on using appropriate reagents to distinguish Ig classes in a multiplex format. Alternatively, a bridging assay using labeled capsids as secondary reagents can detect all capsid-bound antibody classes (Figure 3B).^48^^,^^69^

The selection of rAAV capsid-derived reagents is an essential consideration for TAb assays. These vary from assay to assay, ranging from intact rAAV vectors or a suitable surrogate, such as empty capsids or capsid proteins.^48^ Capsid protein (or protein fragments) may not represent all relevant epitopes or conformational epitopes on the capsid surface; thus, using intact capsids is recommended over surrogates. Moreover, empty capsids carry different charges compared with intact capsids, which may affect their antibody-binding affinity,^70^ although the evidence for this is limited.^48^

### Assay cutoffs, screening titer cutoffs, readout and quality control of anti-AAV antibody assays

**Figure 4:**
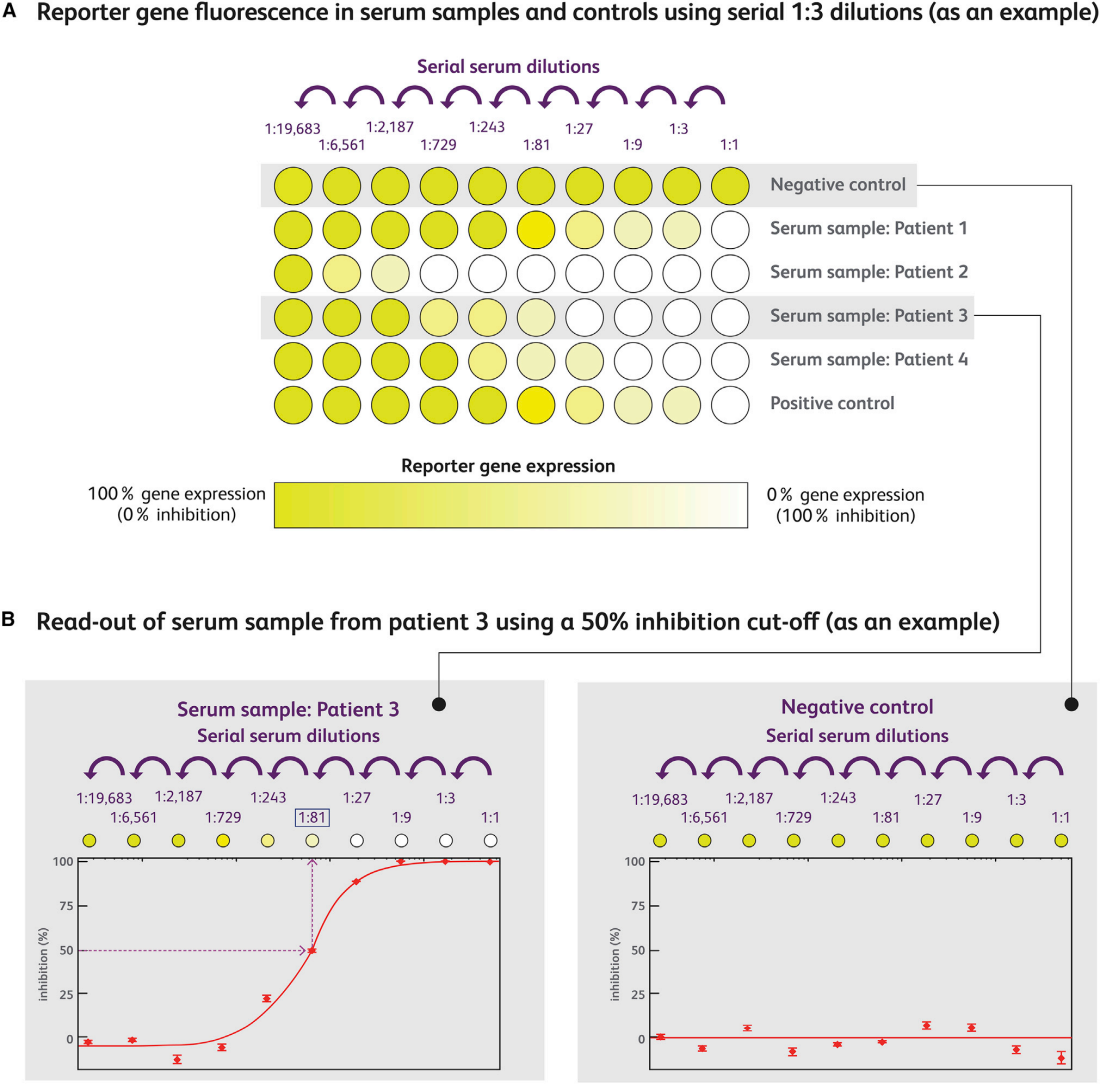
Readout of a semi-quantitative transduction inhibition assay These principles also apply to total antibody assay. (A) Reporter gene expression in serial one in three dilutions of serum samples of four patients and positive and negative controls. The negative control does not contain neutralizing anti-AAV antibodies (or non-antibody neutralizing factors); therefore, the rAAV vector transduces the target cells where the reporter gene is expressed (yellow well). The positive control contains neutralizing anti-AAV antibodies, often marginally above the screening titer cutoff. Patient serum samples one to four contain varying levels of neutralizing anti-AAV antibodies (or non-antibody neutralizing factors) that affect the level of reporter gene expression seen at different dilutions. (B) The degree of inhibition of reporter gene expression is plotted against the dilutions of the serum sample.^48^^,^^49^^,^^56^ The 50% inhibition cutoff from the resulting curves is based on the highest sample dilution that achieves 50% inhibition of vector transduction in comparison with the negative-control sample. In this example, patient 3 has an anti-AAV NAb titer of 1:81, which will be compared with the screening titer cutoff to determine patient eligibility. If the patient 3 titer of 1:81 ≥ screening titer cutoff (e.g., 1:3), the patient would be ineligible for the rAAV GTx. AAV, adeno-associated virus; rAAV, recombinant AAV; TAb, total antibody; TI, transduction inhibition.

TI and TAb assays may be run semi-quantitatively, using a serially diluted patient sample, or qualitatively, using a single predefined dilution. In late-phase clinical studies with more advanced protocols, serial dilutions are commonly used to enable the reporting of anti-AAV antibody titers semi-quantitatively (Figure 4). The assay cutoff is a protocol-specific parameter above which a sample dilution is reported as positive for neutralizing activity.^56^ Assay cutoffs vary between methods, and are set at a defined threshold denoting a positive sample (e.g., 50% TI). Screening titer cutoffs to determine patient eligibility are defined at a prespecified dilution (e.g., ≥1:4). They are typically established using animal studies that compare transduction in naïve versus seropositive animals. However, as cohorts in large-animal studies (e.g., in dogs, pigs, macaques) are often small, and translatability of animal data to humans is often limited, establishing appropriate screening titer cutoffs is complex.^56^ Other methods used to assign screening titer cutoffs are described elsewhere.^71^ Figure 4 shows a semi-quantitative TI assay (principles also apply to TAb assays), using an example with serial 1:3 dilutions and a 50% inhibition cutoff.

Quality and functional assessments of TI and TAb assays require PC and NC samples.^48^^,^^49^ Often the PC titer is chosen to be marginally higher than the screening titer cutoff.^71^ The PC may consist of polyclonal or monoclonal serotype-specific anti-AAV antibodies and may use commercially available proprietary reagents to facilitate cross-product data comparison.^48^ Either serotype-specific or cross-reacting PC reagents are used to characterize assay performance parameters, including sensitivity, precision, selectivity, and robustness.^48^ The choice of PC reagent should be carefully considered, as some monoclonal PCs may provide strict serotype specificity, while others, including polyclonal PCs, cross-react with multiple capsids.^48^ The NC typically comprises pooled sera from several NAb-negative individuals or naïve animals^49^ and is used to monitor assay performance^49^; it is also used as a diluent, and to normalize signals.^48^ Monoclonal antibodies with unique or narrow specificity may also serve as an NC; for example, anti-AAV6 mouse monoclonal antibodies (ADK6) could be used as an NC for an AAV9 assay and, reciprocally, anti-AAV9 mouse monoclonal antibodies (ADK9) could be used as an NC for an AAV6 assay. The main challenge in developing NCs is the high prevalence of pre-existing antibodies, so a confirmatory step may be warranted to exclude antibody-positive samples.^49^

### Comparison of assay types and general considerations

There is an absence of standardization within and between TI and TAb assays. Therefore, an accurate comparison of assay performance and clinical utility is currently not feasible.^48^^,^^49^^,^^72^ An assay that can determine low-level anti-AAV antibodies is ideal, but there may be a trade-off between assay characteristics, such as complexity, sensitivity, and specificity. The advantages and potential disadvantages of both assay types are described in Table 1. They should be considered when developing a bioanalytical strategy for a particular rAAV GTx in context with the specific vector construct, the dose, the mode of administration, the disease indication, and the patient population. The paucity of head-to-head data correlating anti-AAV antibody results with clinical outcomes makes it challenging to recommend a single approach. In addition, the lack of reference reagents for TI and TAb assays is recognized as an unmet need in the field.

**Table 1:** Comparison of transduction inhibition and total antibody assays

Assay	TI assay	TAb assay
Principle	• Cell-based assay that uses rAAV vector encoding a reporter gene^49^	• Immunoassay (e.g., ELISA or ECLA) capture-based method^48^
	• Measures the ability of plasma or serum samples containing NAbs and non-antibody neutralizing factors to reduce the transduction of cells^49^	• Measures the presence of anti-AAV antibodies (regardless of their neutralizing activity)^48^
Advantages	• Directly measures TI by detecting both NAbs and non-antibody neutralizing factors that may impact transduction^48^^,^^49^	• Less complex to implement and automate than a TI assay,^48^ and potentially could be developed into test kits for implementation across multiple clinical laboratories^48^
	• Common design using the same rAAV capsid as GTx with choice of reporter genes and cell lines^49^	• Antigen-capture format can be used to detect specific immunoglobulin isotypes^48^
Potential disadvantages	• Requires specialist laboratories and expertise	• Does not specifically predict TI^48^
	• More challenging to be developed into commercialized test kits	• Variety of platforms and format options makes standardization challenging^48^
		• Potential detection of low-avidity cross-reactive Abs that may not have relevance for successful GTx^48^

AAV, adeno-associated virus; ECLA, electrochemiluminescence assay; ELISA, enzyme-linked immunosorbent assay; GTx, gene therapy; NAb, neutralizing antibody; rAAV, recombinant AAV; TAb, total antibody; TI, transduction inhibition.

Significant challenges encumber the standardization of anti-AAV antibody assay results for the diversity of rAAV GTx programs using the same or antigenically related capsid serotypes. Standardization and commercialization are complex, owing to heterogeneity in cell lines, the lack of harmonized analysis, and the associated costs and hurdles intrinsic to upscaling inherently intricate assays in a rare disease setting.^49^ Selection of a screening assay in early development warrants careful consideration since the characteristics of the study subject population may change if the assay methodology is changed in late-stage development. Although some sponsors of late-stage clinical trials may be wary of implementing TI assays due to the complexities involved with the setup, ongoing or recently completed clinical trials have successfully used robust and reliable TI assays that are suitable for routine clinical testing.^33^^,^^71^^,^^73^^,^^74^^,^^75^

Several studies indicate a reasonable correlation between TAb and TI results for some serotypes (AAV1, AAV3B, AAV5, and AAV8) but not for others (AAV9, AAVrh74, and AAVDJ).^19^^,^^40^^,^^76^^,^^77^^,^^78^ However, a study involving healthy volunteers using both TAb and TI assays for AAV5 found that a subset of individuals was positive in one assay and negative in the other.^41^^,^^62^ Generally, in samples with high NAb titers, concordant assay results (positive) would be expected between TI and TAb assay types.^19^ To resolve discordance between TI and TAb assays, a dual-assay screening strategy has been proposed for some rAAV-based GTx trials to identify individuals who are negative for both TAbs and NAbs and who may be more likely to respond to GTx.^41^^,^^62^ However, this approach is likely impractical given the difficulties with standardizing assays, as mentioned above. Also, having eligibility requirements based on both TI and TAb assay may further limit clinical trial candidates. Another approach could include the generation of pre-clinical data establishing the relationship between these two platforms using well-characterized and robust TI and TAb assays.

### Companion diagnostics

Multiple regulatory guidelines relevant to rAAV gene therapy recommend that sponsors consider the concurrent development of diagnostic tests to screen for pre-existing anti-AAV antibodies.^79^^,^^80^^,^^81^ If the test is considered essential for safety and/or efficacy and the eligible GTx clinical trial candidates experience favorable treatment outcomes, such tests may be classified as a companion diagnostic (CDx).^48^^,^^49^ The formal regulatory approval of an anti-AAV antibody assay as a CDx will reflect that it is robust, sufficiently sensitive, specific to the anti-AAV antibodies in question, and appropriate for the specific rAAV GTx.

Therefore, the submission of the marketing application for the CDx and the biologics license application for the rAAV GTx should be coordinated to support contemporaneous marketing authorizations. For the co-development of a CDx with an rAAV GTx, the sponsor must define the assay’s use and its respective risks and benefits; also the patient population(s) that would benefit from using the assay in conjunction with therapy need to be defined.^82^ Ideally, the CDx and the GTx should be co-developed contemporaneously early on so the assay is ready for early clinical studies, eliminating the need for potential bridging studies later on. The assay needs to be appropriately developed under applicable quality and industry standards.^81^^,^^82^

The regulatory environment for CDx tests for rAAV GTx is evolving and to some extent, open to interpretation: For Onasemnogene abeparvovec (spinal muscular atrophy type I), which has been licensed by the Food and Drug Administration (FDA) (2019),^83^ European Medicines Agency (EMA) (2020),^84^ and the Japanese Ministry of Health, Labour and Welfare (2020),^85^ anti-AAV TAb titers are measured with a laboratory-developed test in the United States and Europe, and a CDx in Japan.^86^ For Valoctocogene Roxaparvovec (hemophilia A), which has been licensed by EMA (2022),^87^ a CE-marked TAb assay is available as a CDx under the European In Vitro Diagnostic Directive (IVDD).^88^ For etranacogene dezaparvovec (hemophilia B), which has been licensed by the FDA at the time of writing this manuscript (December 2022),^89^ one of the FDA post-marketing requirements is to validate a sensitive and accurate TI assay for the detection of anti-AAV5 NAbs up to titers of 1:1,400 or higher (currently, at the time of FDA approval, there is no validated anti-AAV5 NAb assay for etranacogene dezaparvovec available).^90^ The currently licensed rAAV GTx products that are administered through non-systemic routes of administration do not use CDx tests to determine anti-AAV antibodies^91^^,^^92^ (in line with the notion that some non-systemic routes of administration may be less affected by anti-AAV antibodies, see section ‘Overview of anti-AAV antibodies’).

In summary, the regulatory landscape regarding CDx for rAAV GTx is evolving, and careful consideration is necessary to develop an appropriate CDx strategy. Early consultation with the regulatory agencies on the topic is highly recommended.

### Implications of anti-AAV antibodies for efficacy, safety, and GTx eligibility

Previous rAAV GTx studies in animals^27^^,^^93^^,^^94^^,^^95^ and humans^96^^,^^97^^,^^98^^,^^99^ demonstrated that pre-existing anti-AAV NAbs can limit or completely block transgene expression even at low titers. In animal studies, undetectable factor IX (FIX) levels were reported in rAAV8-treated mice that had been inoculated with anti-AAV8 NAbs,^27^ and a 96% loss of FIX expression was observed in the presence of AAV2 NAb titers of just 1:3.8 in rAAV2-treated mice^94^; however, the impact of pre-existing NAbs was found to vary depending on the serotype.^93^^,^^94^^,^^97^ In macaques, an almost complete block of transduction was reported with AAV8 NAbs >1:5.^95^ Similarly, in another macaque study, the complete absence of FIX expression was demonstrated with AAV8 NAb titers of 1:5.^93^ In contrast, a study investigating the effect of AAV9 Abs on transduction efficiency in macaques reported that anti-AAV9 TAb titers up to 1:400 did not block hepatic gene transfer.^100^ An important consideration is that the first two macaque studies used TI assays, whereas the third study used a TAb assay to determine anti-AAV antibody titers. As stated earlier, meaningful comparisons of titers across assays are nearly impossible to make.

In humans, FIX expression was attenuated in a patient with pre-existing NAb titer of 1:17 who was administered an AAV2 vector to treat hemophilia B.^96^ In another study, which evaluated rAAV (Spark100) GTx in hemophilia B patients, George et al. (2017) reported that the single participant with an anti-AAV-Spark100 NAb titer of 1:1 achieved lower FIX activity than participants with lower NAb titers (<1:1).^99^

It is generally accepted that antibodies mediate innate and B and T cell-dependent immune responses; for example, pre-existing antibodies can bind rAAV vectors and redirect them to secondary lymphoid organs^101^ to be taken up by antigen-presenting cells.^101^^,^^102^ This could change the biodistribution profile and clearance of the rAAV vectors, potentially resulting in a lower distribution to target cells and/or inflammation.

Neutralizing or non-neutralizing IgM/IgG antibodies are believed to form immune complexes with circulating vectors, which can activate the complement system via the classical pathway.^103^ Activation of the complement system can lead to thrombotic microangiopathy (TMA) syndrome and kidney injury.^104^^,^^105^^,^^106^^,^^107^^,^^108^ In an rAAV GTx study in DMD patients, three participants developed complement activation following high-dose vector administration (2E14 vg/kg).^103^^,^^109^^,^^110^ However, as all patients had been seronegative for both TAbs and NAbs at baseline, neither TI nor TAb assays would have predicted the complement activation in these cases.^110^ Innate immune responses such as complement activation may result from the primary immunization to the capsid in naïve patients upon rAAV GTx administration and depend on the administered vector dose (mostly seen with high doses >1E14 vg/kg).^84^^,^^109^^,^^111^ *De novo* IgM antibody formation in the days after rAAV GTx administration may better predict complement activation and TMA-like adverse events than the antibody status at baseline. As rAAV GTx gathers momentum, increased efforts are under way to understand and manage adverse events associated with host immune responses by intensifying patient monitoring, modifying patient-eligibility criteria for clinical trials, and adjusting immunosuppressive regimens.^112^^,^^113^

Not all clinical trials have used AAV seropositivity as an exclusion criterion. The phase 3 study of etranacogene dezaparvovec in hemophilia B patients used an AAV5 vector and treated patients independent of their anti-AAV NAb status^38^ using an unvalidated clinical trial assay.^90^ The patient subgroup with detectable pre-existing neutralizing anti-AAV5 antibodies ≤1:678 showed mean FIX activity that was numerically lower compared with the patient subgroup without detectable pre-existing neutralizing anti-AAV5 antibodies. Patients with and without pre-existing anti-AAV5 NAbs demonstrated hemostatic protection. One trial participant with a pre-existing AAV5 NAb titer of 1:3212 did not respond to therapy (no FIX expression was observed), and FIX prophylaxis was restarted.^38^ Currently, limited data are available to offer clear hypotheses for these results. At present, therapeutic levels of transgene expression need to be achieved with the first dose of rAAV GTx. Systemic re-administration of the same and likely other rAAV vector serotypes will not be possible because of the strong, durable, and cross-reactive antibody response that manifests after the first dose of rAAV GTx. Preventive strategies to potentially circumvent this issue in the future are currently being investigated in clinical trials.^114^

### Investigative strategies to overcome the effect of pre-existing anti-AAV antibodies

Currently, a significant subset of patients who exhibit pre-existing NAbs is likely to be excluded from GTx clinical trials or treatment. Consequently, various strategies to overcome this challenge are being explored. These are broadly divided into GTx-related and pre-treatment approaches,^72^^,^^115^ as summarized in Table 2.

**Table 2:** Investigative strategies to mitigate the effect of pre-existing anti-AAV antibodies in patients

Potential approach: rationale	Considerations and examples
*GTx-related approaches*	
Direct delivery to target organ: minimize exposure to NAbs	• Minimizes systemic exposure to the vector^43^^,^^47^^,^^115^^,^^116^^,^^118^
	• This option is not suitable for all target tissues, and some administration routes may elicit strong T cell responses against the transgene^115^
	• Example: Subretinal delivery of AAV2-hRPE65v2 in five patients with Leber congenital amaurosis type 2 resulted in improvement in visual acuity sustained for 3 years^115^
	• Example: Saline flushing of the liver followed by injection into the portal vein allowed efficient human FIX transduction by AAV8 in non-human primates with NAb titer ≤1:56^118^
Administer high dose: overcome NAbs	• May be effective in the presence of low-titer (1:5–1:17) NAbs^93^^,^^96^
	• The higher vector load could trigger an anti-capsid T cell response^96^^,^^136^, which is a potential safety risk, particularly in the setting of pre-existing humoral response
	• Example: Tenfold increase in vector dose partially overcame low-level pre-existing antibodies to AAV in mice models^137^
Administer empty capsids: adsorb anti-AAV antibodies	• Advantages include the lack of pharmacological intervention and the ease of manufacturing empty capsids (as a by-product of AAV vector production)^116^
	• May lead to greater viral load, potentially triggering an anti-capsid T cell response^102^, particularly in the setting of pre-existing humoral response
	• Example: Adding empty capsids dose-dependently to adsorb anti-AAV antibodies, successfully facilitating transduction in murine and non-human primate models – even at high titers. Moreover, mutated capsids were reported to adsorb antibodies without entering the target cell^117^
Modify capsid/switch AAV serotype or engineering/cloaking of AAV: reduce capsid susceptibility to NAbs	• Coupled immunomodulation via cloaking is technically feasible, although such a strategy remains challenging in terms of engineering and manufacturing the capsid^115^^,^^119^
	• Engineering capsid could deleteriously modify tissue tropism and increase cross-reactivity among AAV serotypes^47^^,^^116^^,^^138^
	• Example: Engineered AAV vectors elicited reduced immune responses and enhanced gene expression across different tissues, including liver, muscle and retina in clinically relevant mouse and pig models^119^
*Pre-treatment approaches*	
Immunosuppressive drugs: prevent/eradicate humoral immune response to AAV	• Several B and T cell depletion therapies are available^120^^,^^139^
	• Potential risks are associated with incomplete eradication of pre-existing high-titer NAbs with systemic immunosuppression, such as rituximab^116^^,^^140^
	• Example: Rituximab reduced anti-AAV NAb titers in rheumatoid arthritis patients with titers ≤1:1000, although only a minority dropped to <1:5^16^
	• Example: In a patient with Pompe disease, treatment with rituximab and sirolimus mitigated an immune response to an AAV1-GAA vector, which may allow for repeat administration in the future^121^
Plasmapheresis: selective depletion of anti-AAV IgGs	• Has been shown to reduce antibody titers^64^^,^^122^
	• Potential risks are associated with incomplete eradication of pre-existing high-titer NAbs^123^
	• Example: Frequent sessions of plasmapheresis resulted in reduction of NAbs specific for AAV1, 2, 6, and 8 to undetectable levels or titers <1:5 in seropositive patients (mainly when initial titers were ≤1:20)^123^
IgG cleaving enzymes from certain *Streptococcus* species: inhibiting an IgG immune response	• IdeS (from *Streptococcus pyogenes)* treatment has thus far been shown to have a favorable safety profile in solid organ transplant recipients^141^
	• Has no impact on IgA, IgM, IgD, and IgE antibodies^142^
	• Potential alternatives include IdeZ from *Streptococcus equi* subsp. *zooepidemicus*^125^
	• Example: IdeS treatment before rAAV vector infusion resulted in enhanced liver transduction in non-human primates, even in the setting of vector re-administration, and reduced anti-AAV antibody levels from human plasma samples *in vitro*, including plasma from prospective GTx trial participants undergoing GTx for Crigler–Najjar syndrome^124^
Anti-FcRn antibodies: reduce IgG levels	• FcRn1 helps maintain circulating IgG levels, and its inhibition can potentially reduce NAb titers by ∼80% for 60 days^126^
	• Has no impact on IgA, IgM, IgD, and IgE antibodies^143^
	• Example: Anti-FcRn antibodies lowered and maintained the reduction of total IgG and IgG subclasses following multiple subcutaneous injections in healthy adults^127^

AAV, adeno-associated virus; FcRn, neonatal crystallizable fragment receptor; GAA, acid α-glucosidase; GTx, gene therapy; IdeS, IgG-degrading enzyme of *Streptococcus pyogenes*; IgA, immunoglobulin A; IgD, immunoglobulin D; IgE, immunoglobulin E; IgG, immunoglobulin G; IgM, immunoglobulin M; NAb, neutralizing antibody.

GTx-related approaches include using higher GTx doses,^72^^,^^116^^,^^117^ co-administering empty “decoy” capsids,^72^^,^^97^^,^^115^^,^^116^ local delivery to the target organ or tissue,^43^^,^^47^^,^^115^^,^^116^^,^^118^ use of alternate capsids, or cloaking the rAAV capsid.^72^^,^^115^^,^^119^ These strategies aim to limit immune clearance of the vector and reduce susceptibility to NAbs. Pre-treatment-related strategies are designed to suppress the humoral immune response by reducing antibody production (for example, by using immunosuppressive drugs),^16^^,^^47^^,^^97^^,^^116^^,^^120^^,^^121^ or by reducing the levels of serum IgG by plasmapheresis,^47^^,^^115^^,^^116^^,^^122^^,^^123^ preconditioning with IgG-degrading enzyme of certain *Streptococcus* species (IdeS or IdeZ),^124^^,^^125^^,^ and neonatal crystallizable fragment receptor (FcRn) inhibition.^126^^,^^127^ Ultimately, a combination of alternative AAV variants, alternate routes of administration with minimal immune exposure, and techniques to reduce anti-AAV NAb levels by physical methods or pharmacological modulation of the humoral immune response may be needed to overcome the impact of pre-existing AAV NAbs in patients who would otherwise not be eligible for AAV-mediated GTx.^47^ Timing, dosing, and administration of immune modulation approaches, such as IdeS, must be tailored to specific AAV serotypes to optimize transduction.^128^^,^^129^

The main anticipated use of rAAV GTx is as a single treatment; high and persistent NAb levels after vector administration are expected to make re-administration particularly challenging. However, re-administration of rAAV GTx may be necessary in some clinical scenarios, such as early-onset myopathy or in tissue compartments with higher turnover. Preventive strategies are being explored as adjunctive therapies to reduce NAb formation and to enable re-dosing at a later time point, with encouraging preliminary findings.^124^^,^^130^^,^^131^^,^^132^^,^^133^^,^^134^ To enable re-administration of rAAV GTx, a combination of approaches might be required to reduce NAb levels sufficiently or to provide preventive inhibition of antibody formation with first exposure.^115^

### Conclusions

Accurate and robust detection of anti-AAV antibodies prior to systemic GTx administration is an important consideration regarding the efficacy and safety of the therapy. Conservative eligibility criteria (including overly stringent screening titer cutoffs) could exclude a patient from a potentially transformative or life-saving treatment. Conversely, overly lenient inclusion criteria could render treatment ineffective or have potential safety implications. Currently, no universal method exists to determine clinically relevant antibody levels. Either TI or TAb assays are used, both of which have advantages and potential disadvantages. Broadly speaking, the TI assay focuses on the clinically relevant parameter of transduction inhibition, and therefore provides a direct measure of antibodies that could impact the outcome of rAAV GTx. However, it requires more expertise and effort to become established in routine clinical use. In contrast, the TAb assay detects both NAbs and non-NAbs and is less complex to implement and automate. Formal regulatory approval of anti-AAV antibody assays as CDx tests will confirm their suitability for the specific rAAV GTx. During the development of the bioanalytical strategy for a particular rAAV GTx, the characteristics of the two assay types should be considered in the context of the specific vector construct, vector dose, mode of administration, disease indication, patient population, and patient population size.

Standardization is hard to achieve within and between TI and TAb assays for several reasons, including but not limited to a lack of standardization of critical assay components, differences in sample handling/processing, and a lack of harmonized analytical procedures. Therefore, comparing anti-AAV antibody data generated across different rAAV GTx programs is highly challenging. Detailed reports of assay parameters alongside the results will be helpful for the community to apply learnings across programs. In addition, it is anticipated that interlaboratory programs that compare and analyze the critical quality attributes of viral vectors and that develop physical reference materials could improve measurement consistency across the industry.^135^

The presence of pre-existing NAbs against AAV can have significant consequences for the affected patients and their families. A seropositive patient might be ineligible to receive a potentially transformative treatment, while a patient who tested negative for NAbs still faces the potential risk of seroconverting prior to GTx administration (e.g., during the run-in phase or the delayed treatment arm of a clinical trial, or between eligibility testing and dosing for a licensed GTx). Research is ongoing to provide potential solutions for patients with anti-AAV antibodies who are currently excluded from receiving rAAV GTx.
